# Is Linear Wound Closure Acceptable Option for Congenital Midline Cervical Cleft Excision in Neonates?

**Published:** 2013-07-09

**Authors:** Xenophon Sinopidis, Antonios Panagidis, Vasileios Alexopoulos, George Georgiou

**Affiliations:** Department of Paediatric Surgery, University of Patras, Patras 26504, Greece and Department of Surgery, Karamandanion Children’s Hospital, 26331 Patras, Greece; Department of Surgery, Karamandanion Children’s Hospital, 26331 Patras, Greece; Department of Surgery, Karamandanion Children’s Hospital, 26331 Patras, Greece; Department of Surgery, Karamandanion Children’s Hospital, 26331 Patras, Greece

**Dear Sir,**

 We read with interest a case report on a rare anomaly “congenital midline cervical cleft” published in this journal.[1] We wanted to share our reflections on the choice of wound closure technique. We agree that z-plasty is a good technique for late presenting cases owing to grown-up and more rigid lesion. However we have applied a linear closure after excising congenital midline cervical cleft in a neonate. We were concerned that this would possibly result in a bad scar or contracture with significant aesthetic and functional impairment. Nevertheless, at the age of 19 month, the patient has a cosmetic wound, permitting full extension of the neck without webbing (Fig. 1).[2] We believe that the most important factor for this result is the timing of operation. Early treatment of the lesion at the neonatal age is crucial for an optimal cosmetic and functional result. During this period the lesion has the smaller size encountered, and wound healing is excellent. Postoperatively the infant is more malleable to neck extension exercises, and application of scar healing ointments is easier. We believe that in case of delayed presentation for treatment, certain factors (lesion size, wound healing process, patient manipulation) might get worse, and finally z-plasty renders the preferable option for wound closure.

**Figure:**
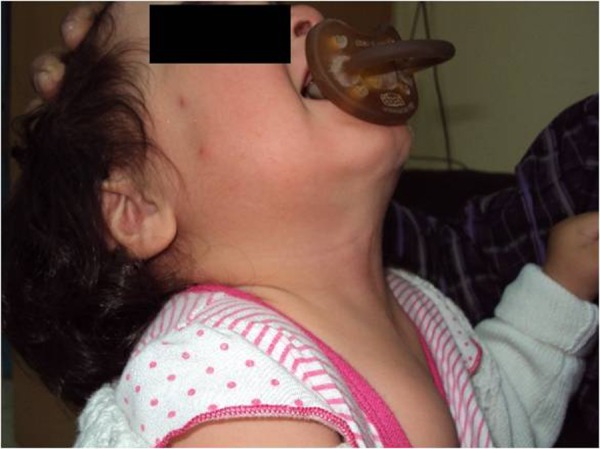
Figure 1: Long term cosmetic and functional result in a female patient operated for congenital midline cervical cleft as a neonate.

## Footnotes

**Source of Support:** Nil

**Conflict of Interest:** None declared

